# The 4^th^ and 5^th^ Annual U.S. World Hospice and Palliative Care Day conferences: Unifying the global palliative care community

**DOI:** 10.1017/S1478951525100291

**Published:** 2025-07-18

**Authors:** William E. Rosa, Shila Pandey, Andrew S. Epstein, Liz Blackler, Lauren Akua Koranteng, Dana Greenfield, Paul Yoon, Craig D. Blinderman, Judith E. Nelson, William S Breitbart

**Affiliations:** 1 Department of Psychiatry and Behavioral Sciences, Memorial Sloan Kettering Cancer Center, New York, NY, USA; 2 APP Professional Development Department, Supportive Care Service, Memorial Sloan Kettering Cancer Center, New York, NY, USA; 3 Department of Medicine, Memorial Sloan Kettering Cancer Center, New York, NY, USA; 4 Weill Cornell Medical College, New York, NY, USA; 5 Ethics Committee, Memorial Sloan Kettering Cancer Center, New York, NY, USA; 6 Department of Pediatrics, Memorial Sloan Kettering Cancer Center, New York, NY, USA; 7 Department of Spiritual Care, Memorial Sloan Kettering Cancer Center, New York, NY, USA; 8 Supportive Care Service, Memorial Sloan Kettering Cancer Center and Weill Cornell Medical College, New York, NY, USA; 9 Supportive Care and Critical Care Services, Memorial Sloan Kettering Cancer Center and Weill Cornell Medical College, New York, NY, USA

**Keywords:** Palliative care, supportive care, global palliative care, World Hospice and Palliative Care Day, virtual conference

## Abstract

**Objectives:**

On October 3–4, 2023 and September 30–October 1, 2024, the Memorial Sloan Kettering Cancer Center Department of Psychiatry and Behavioral Sciences and Supportive Care Service hosted the 4^th^ and 5^th^ Annual U.S. Celebration of World Hospice and Palliative Care Day (WHPCD) conferences, respectively. This article describes both events and lessons learned in anticipation of the 6^th^ annual conference to be held October 6–7, 2025.

**Methods:**

The 4^th^ and 5^th^ annual events, conference planning team reflection, and attendee evaluation responses are summarized.

**Results:**

Since 2020, the conference has attracted attendees from around the world. Two primary aims continue to guide the event: community building and wisdom sharing at the intersection of art and science. Both the 2023 and 2024 events consisted of 13 unique interactive sessions addressing diverse hospice and palliative care topics delivered by interprofessional experts in palliative care (43 faculty in 2023 and 54 in 2024). Multidisciplinary registrants more than doubled from 764 in 43 countries (2023) to 1678 in 87 countries (2024). Complimentary registration for colleagues in low- and middle-income countries (LMIC), students and trainees, and individuals experiencing financial hardship remains a cornerstone of inclusion and equitable access to the event.

**Significance of results:**

The U.S. WHPCD Conference provides a virtual platform to disseminate high-quality science, honor both clinician and patient and caregiver experiences, and celebrate hospice and palliative care delivery during substantial local and global change across practice and policy domains. We remain committed to ensuring an internationally relevant, culturally diverse, and multidisciplinary and interprofessional agenda that will draw increased participation worldwide during future annual events.

The 4^th^ and 5^th^ Annual U.S. Celebration of World Hospice and Palliative Care Day (WHPCD) conferences were held on October 3–4, 2023 and September 30–October 1, 2024, respectively, co-sponsored the Department of Psychiatry and Behavioral Sciences and Supportive Care Service of Memorial Sloan Kettering Cancer Center (MSK). Building on the promising outcomes of the first 3 conferences (2020–2022) (Rosa et al. 2022, 2023a, 2021), the 4^th^ and 5^th^ annual conferences used a live virtual format, condensed over 2 half-days with alternating times to increase global access. We aim to provide a brief report of these events in preparation for the 6^th^ Annual U.S. Celebration of WHPCD on October 6–7, 2025.

The 2023 conference attracted 764 registrants from 43 countries; registration more than doubled for the 2024 conference with 1678 registrants from 87 countries – the most attended Continuing Medical Education (CME) event at MSK to date. Live virtual attendance also more than doubled from 367 unique participants in 2023 to 900 in 2024. Live attendance numbers may be underestimated since several participants logged in as a group to watch the conference together. Registrants who did not attend the conference live had the option to view recorded sessions on demand.

The conference course directors and planning committee were comprised of an interprofessional team representing both pediatric and adult palliative care in nursing (W.E.R. and S.P.), medicine (A.S.E. and D.G.), spiritual care (P.Y.), social work (L.B.), bioethics (W.E.R. and L.B.), pharmacy (L.A.K.), anthropology (D.G.), and a member of the Patient and Family Advisory Council for Quality. The format of the conference remained virtual to increase access to international participants and amplify the voices and priorities of colleagues working in low- and middle-income countries (LMICs). Over these 2 years, we expanded CME credits to include social work credits in addition to the previously available credits for physicians, nurses, psychologists, and advanced practice providers. Chaplains certified by the Board of Chaplaincy Certification Inc. could use the program toward their continuing education requirements. For both years, we were able to offer complimentary registration through a generous anonymous donor, covering the costs for all registration, CME, technology, and honoraria. All conference registrants receive access to session recordings and PDF slides indefinitely on the CME portal.

## A virtual coming together unifying collaborators, countries, and contexts

The Worldwide Hospice Palliative Care Alliance (WHPCA) launched WHPCD in 20025 as an annual unified day of action to celebrate and support hospice and palliative care globally. For our conference, the themes of WHPCD are integrated into conference planning to recognize global palliative care trends. The 2023 theme for WHPCD was *Compassionate Communities Together for Palliative Care* (Worldwide Hospice Palliative Care Alliance 2023)and for 2024 it was *Ten Years Since the Resolution: How Are We Doing?* (Worldwide Hospice Palliative Care Alliance 2024). See Table 1 for the 2023 and 2024 WHPCD campaign key messages and asks (action items). The 2023 and 2024 events each consisted of 13 interactive sessions addressing diverse hospice and palliative care topics delivered by interprofessional experts (43 faculty in 2023 and 54 in 2024). Topics covered can be found in Tables 2 and 3.

**Table 1.** Campaign key messages and asks: World Hospice and Palliative Care Day 2023–2024

2023		2024	
Campaign key messages	Key ask	Campaign key messages	Key ask
Compassionate communities as a way to complement existing palliative care services. Compassionate communities promote a holistic definition of health. Compassionate communities are rooted in a health promotion approach to palliative care.	We call upon governments, policymakers, health professionals, community activists, volunteers, people with palliative care needs, and others to come together to encourage the growth of compassionate communities and cities, linked with palliative care all over the world. Become a Compassionate City and join the Compassionate Communities Movement to engage communities as equal partners in delivering quality healthcare at the end of life.	Develop, strengthen, and implement palliative care policies to support the comprehensive strengthening of health systems. Provide basic support, including through multisectoral partnerships. Review and, where appropriate, revise national and local legislation and policies for controlled medicines, with references to WHO policy guidance.	Don’t let palliative care progress fall further behind. Ensure that the palliative care resolution is effectively realized over the next decade. Demand that governments include palliative care and relief of suffering in plans for universal health coverage.

*Source*: Worldwide Hospice Palliative Care Alliance. (2023). Campaign toolkit: compassionate communities together for palliative care. Available at: https://thewhpca.org/resource_category/world-day-toolkit/.

Reprinted with permission from the Worldwide Hospice Palliative Care Alliance.

**Table 2.** The 4^th^ Annual U.S. Celebration of World Hospice and Palliative Care Day conference schedule and session topics.

Session title	Topics addressed
Care, Compassion, and Community	Annual World Hospice and Palliative Care Day Lectureship: state of palliative care research in LMICs, re-humanizing serious illness care, and challenges in delivering community palliative care for people living in poverty
Weaving the Global Narrative of Palliative Change Agents on the Ground	Showcase of palliative care advocacy and leadership across diverse settings: Sudan, Jamaica, Uganda, and Mexico. Barriers and opportunities of each geographic location were discussed.
Transitional Bereavement Care	Breakout session: discussion of approaches to improve transitional bereavement care and the need to build bridges between hospice and palliative care programs and the community to bolster compassionate community-based support for grievers.
Approaches to Community-Based Palliative Care Provision	Breakout session: discussion of barriers and opportunities to achieve goal-concordant care, especially in racial and ethnic minorities. Describes results from a study on the impact of faith language in advance care planning and advance directive documents.
Clinician-Patient Conversation	Interview conducted by a palliative care oncologist with a patient discussing themes such as navigating uncertainty of a serious illness while attempting to maintain an acceptable quality of life, elevating the patient’s voice, and broader efforts to advocate for patients and families.
Top Ten Tips Palliative Care Clinicians Should Know	Behavioral pain management for persistent pain: biopsychosocial framework and nonpharmacological approaches to management of pain in the setting of serious illness. Anorexia/Cachexia: multimodal management of cachexia with interprofessional collaboration. Attention to screening, diagnosis, nutritional support, exercise, education, and psychosocial support were discussed.
Palliative Justice for Minoritized and Marginalized Peoples	Four faculty panel presentations on: advancing justice for all through value-based healthcare; inclusion of LGBTQ+ persons in palliative care; and communication needs of racially minoritized patients.
The Palliative Story Exchange	A storytelling intervention in which conference attendees shared personal stories and reflections in effort to build community, decrease isolation, and rediscover the shared meaning of working in palliative care.
Clinician-Caregiver Conversation	Exploration of a caregiver’s experience with a spouse’s terminal illness; addressed decision-making about potentially life-sustaining treatments, medical aid in dying, and takeaways for all interdisciplinary health professionals to consider in the care of patients and their family caregivers with serious illness.
Serious Illness Communication: Advances, Debates, and Next Steps	Breakout session: expansion of a communication training program, VitalTalk, to Japan; acceptability of a serious illness guide in African American patients with cancer; and state of the science on advance care planning and goal concordant care.
Palliative Care for Non-cancer Conditions	Breakout session: clinical experts and researchers presented on palliative care integration in progressive cardiac, pulmonary, and liver diseases. State of the science, opportunities, and future directions for each organ disease were discussed.
Invited Rapid Fire Talks: Compassionate Communities: Together for Palliative Care	Various topics on palliative care in diverse communities: “Rural Appalachia”; “TransTasman Tele-Palliative Care: Challenges and Opportunities for Compassionate Service Delivery”; “Geographic Mapping of Palliative Care Access in Malaysia”; “Palliation for Health Care Workers Through Organizational Compassion”; and “Serving Those Who Serve: Front Lines of Military Palliative Care.”
Listening to the Silent Screams	Annual Dr. Richard Payne Lecture on Equity, Inclusion, and Belonging in Serious Illness: breakdown on how clinicians can value personhood beyond a medical diagnosis, culturally appropriate communication, and importance of addressing spiritual beliefs.

**Table 3.** The 5^th^ Annual U.S. Celebration of World Hospice and Palliative Care Day Conference schedule and session topics

Session title	Topics addressed
Life Until the End: Ubuntu, Culture, and What Truly Matters in Responding to Suffering	Annual World Hospice and Palliative Care Day Lectureship: palliative care program development in Rwanda and other African countries, applications of “ubuntu philosophy” to build compassionate communities, impact of colonization on understanding of death, and importance of culture and metaphors in responding to suffering.
Recognition of the 10-Year Anniversary of the World Health Assembly Resolution on Palliative Care	Highlight of the 10-year anniversary since the World Health Assembly passed the only stand-alone resolution on palliative care, calling for all countries to increase access to palliative care as an essential part of comprehensive, high-quality healthcare.
Access, Equity, and Inclusion in Psychedelic Medicine and Therapies	Breakout session: state of psychedelic-assisted therapy research, lack of diverse backgrounds in the populations studied, and approaches to improve access and recruitment of underrepresented populations while respecting ethical principles of traditional Indigenous medicine.
Care of Children with Serious Neurologic Impairment	Breakout session: palliative care approaches to care of children with serious neurologic impairment, including communication frameworks, respite for caregiver parents, and use of photo and video to restore dignity.
Virtual Schwartz Rounds	Featuring an interdisciplinary team from the University of California, San Francisco (UCSF) discussing a challenging case focused on the emotional and social aspects of patient care and relationship-building, as well as the personal experiences of the physician, nurses, and social workers involved.
Top Ten Tips Palliative Care Clinicians Should Know	Care for Patients with Hematologic Malignancies: review of the current state and barriers to integrate palliative care. Before the Hospital – Partnering with Emergency Medical Services to Improve Serious Illness Care: opportunities to improve end-of-life care by collaborating with emergency medical services including approaches to make sustainable changes on a systems level.
Intentionally Interprofessional Palliative Care	Approaches to promote transdisciplinary practice in which all team members practice to the top of their licensure with curiosity and supportiveness about other clinicians’ unique contributions.
Patient & Caregiver Conversation	Exploration of a patient and caregiver’s experience with a spouse’s terminal illness; addressed variations in coping styles, impact on relationships, role of palliative care, use of formal psychosocial supports, and takeaways for all interdisciplinary health professionals to consider in the care of patients and their family caregivers.
Invited Rapid Fire Talks	“Telehealth-Based Geriatric Assessment and Supportive Care Intervention”; “Advance Care Caregiving in Young Adulthood: Developmental and Intergenerational Challenges”; “The Role of Spirituality in Pain Experiences Among Adults with Cancer”; “Addressing Mental Health in Serious Illness: Towards Integrated Palliative Care”; and “Addressing Challenges with Sedation Practices in End-of-Life Care.”
Leadership Experiences of Women of Color in Palliative Care	Breakout session: 5 interdisciplinary women of diverse backgrounds come together to discuss how their identities have impacted their experience as hospice and palliative care clinicians.
Potentially Non-beneficial Interventions at the End-of-Life	Breakout session: cardiopulmonary resuscitation at the end of life; palliative care integration and imitating potentially non-beneficial care in the intensive care unit in India; role of parenteral nutrition at the end of life; and communication strategies to navigate these complex decisions.
The Palliative Story Exchange	A storytelling intervention in which conference attendees shared personal stories and reflections in effort to build community, decrease isolation, and rediscover the shared meaning of working in palliative care.
Navigating Culture, Race, and Spirituality in Palliative Care	Annual Dr. Richard Payne Lecture on Equity, Inclusion, and Belonging in Serious Illness: utilizing storytelling to describe challenges navigating culture, race, and spirituality in palliative care.

M.R. Rajagopal, MD, delivered the 2023 opening keynote, speaking on the state of palliative care research in LMICs and the advantages of community participation in care. He called out unique challenges for people with serious illness and poverty, reminding us “care” is not charity, rather the right of the patient and family. The 2024 opening keynote featured Christian Ntizimira, MD, MMsGHD, who spoke on his experience during the Rwandan Genocide Against the Tutsi and how this led to his journey into passionate advocacy for palliative care in Rwanda and internationally. Dr. Ntzimira emphasized “the way people die can reflect how the society lives,” discussing the impact of colonization on how cultures understand the meaning of death (Ntizimira et al. 2022; Ntizimira 2023). He also offered applications of Ubuntu philosophy, “I am because we are” (also “I am because you are”), or “humanity towards others,” as a salient guide for practicing engaged and interconnected palliative care.

Following the keynote in 2023, the next session showcased palliative change agents “on the ground,” featuring Dingle Spence, MBBS, Dip Pall Med, FRCR (Jamaica), Nahla Gafer, MD, (Sudan), Eve Namisango, PhD (Uganda), and Felicia Marie Knaul, PhD (U.S./Mexico). Discussions ranged from palliative care needs during the humanitarian crisis in Sudan and how to leverage remote, online care when such communications are available, to Mexico’s approach to develop a national action plan to provide palliative care and pain relief services for those without social security coverage. In 2024, a session celebrated the 10-year anniversary since the World Health Assembly passed the only stand-alone resolution on palliative care, calling for all countries to increase access to palliative care as an essential part of comprehensive, high-quality healthcare (World Health Assembly 2014). Various organizations were represented: Stephen R. Connor, PhD, for WHPCA, Alex Daniels for International Children’s Palliative Care Network (ICPCN), Liliana De Lima, MS, MHA, for International Association for Hospice and Palliative Care (IAHPC), Ednin Hamzah, MD, for Hospis Malaysia, Christian Ntizimira, MD, MMsGHD for African Center for Research on End-of-Life Care (ACREOL), and Kathleen M. Foley, MD from MSK. Faculty discussed the importance of the resolution to globally recognize access to palliation as a basic right; to provide safe medication and good quality palliative care to millions of people around the world; and to address social, cultural, and legal barriers and strengthen capacity at country and regional levels.

Both years provided 2 concurrent breakout sessions on day 1. In 2023, one session featured Wendy Lichtenthal, PhD, FT, and Lauren Breen, PhD, on the medicalization of death, dying, and bereavement care. They emphasized the importance of bereavement-conscious practices, especially during transitions of care (Lichtenthal et al. 2024) and proposed bereavement offerings that should vary based on the level of need rather than a “blanket approach,” creating a vision of a grief-literate society to develop compassionate communities (Breen et al. 2022). The other breakout session featured Danetta Sloan, PhD, MSW, MA, Mrs. Malissa Spaulding, Jennifer Tjia, MD, MS, and Susan DeSanto-Madeya, PhD, RN, on community-based palliative care provision. Drs. DeSanto-Madeya and Tjia discussed the challenges of achieving goal-concordant care for patients living with serious illnesses and provided the Goal Assessment and Prioritization (GAP) Tool as an approach to facilitate goal-concordant communication (DeSanto-Madeya et al. 2025; Fromme et al. 2025). Dr. Sloan and Mrs. Spaulding shared results from a current study on advance care planning outcomes with and without faith language (African American Faith Communities Transcending Dementia).

During one session in 2024, Stacy M. Fischer, MD, reviewed the state of psychedelic-assisted therapy research, specifically with psilocybin. Darron Smith, PhD, PA-C, DFAAPA, discussed the lack of Black, Indigenous, and people of color represented in psychedelic research, and Caroline Dorsen, PhD, FNP-BC, addressed how psychedelics may be used to either lessen or perpetuate marginalization for LGBTQIA+ persons, calling for affirming psychedelic and palliative care spaces. The second breakout featured a discussion of palliative care for children with serious neurologic impairment (SNI). Julie Hauer, MD, a leading expert in the field, provided a framework to discuss the impact of SNI with parents using an iterative language framework that facilitates partnership (Hauer and Wolfe 2014). Jori F. Bogetz, MD, followed with innovative ways to elicit parent narratives and foster collaboration to highlight the challenges and joys of caring for children with SNI (Bogetz et al. 2022). Amy S. Porter, MD, PhD, focused on developing our understanding of “respite” for this population, highlighted the unmet need for their parent caregivers, and described global priorities for pediatric respite research and advocacy (Porter et al. 2024).

Both years featured sessions in which a clinician interviewed a patient and/or family caregiver. In 2023, Andrew Epstein, MD, FAAHPM, spoke with Frank Licciardi, who was treated at MSK and is Chair Emeritus of MSK’s Patient and Family Advisory Council for Quality. They discussed the challenges of deciding where to get cancer treatment, navigating treatment regimens, keeping employment, and trying to maintain “normalcy” while navigating the uncertainty of a serious illness. On day 2 of 2023’s conference, William E. Rosa, PhD, MBE, APRN, spoke with caregiver Amy Hamblin, MA, spouse of the late palliative care pioneer, Dr. J. Randall Curtis. Mrs. Hamblin shared Dr. Curtis’ illness with amyotrophic lateral sclerosis (ALS) and his decision to pursue medical aid in dying at the end of life (Curtis et al. 2024). She explained how they navigated medical decisions in the context of values and beliefs as the ALS progressed. They celebrated the legacy of Dr. Curtis in both critical and palliative care.

In 2024, the patient and caregiver conversations were combined into 1 session on day 2, during which Andrew Epstein, MD, FAAHPM, spoke with an MSK patient, Mr. Leslie Shifrin, and his wife, Mrs. Fran Shifrin. Mr. Shifrin spoke about his experience receiving a diagnosis of pancreatic cancer, challenges navigating treatments, and overcoming anxiety and fears about the future. Mr. Shifrin found comfort engaging with the medical team, utilizing counseling and support groups, and being active in the community. Mr. Shifrin remarked on how he found peace with mindfulness along with being treated as a person – beyond the clinical diagnosis – by the clinical team. Mrs. Shifrin described her shock of the diagnosis as being “hit over the head” and how she learned to build trust with the medical team. She shared limited benefits with formal psychosocial supports and how “digging deep” was painful but, over time, has lessened as they both focus on what matters most.

Both years featured presentations from the *Journal of Palliative Medicine*’s “Palliative Care Specialists Series” (i.e., Top Ten Tips series) moderated by the journal’s Editor-in-Chief, Christopher A. Jones, MD, MBA. In 2023, James Gerhart, PhD, discussed behavioral pain management strategies and reviewed the biopsychosocial framework (Gerhart et al. 2023). He provided an acronym for participants to remember: CARD – compassion, activity, and reduce stress. Dr. Gerhart shared approaches to support family communication about goals of care; being nonjudgmental to non-adherence; encouraging mindfulness, relationship, sleep, paced activity, and stretching; and addressing mental health symptoms. David Blum, MD, PhD, and Barry Laird, MBChB, MD, MRCGP, clarified the differences between anorexia and cachexia, and emphasized the importance of early screening and proactive treatment in high-risk cancers (Blum et al. 2023). They reviewed approaches to assessing functional and inflammatory status in addition to a multimodal approach to treatment, requiring interprofessional clinicians providing nutritional support, exercise, education, and psychosocial support.

In 2024, Neha Kayastha, MD, and Jason A. Webb, MD, DFAPA, FAAHPM, discussed palliative care for patients with hematologic malignancies, including characteristics unique to the population, obstacles to and benefits of palliative care integration, and strategies for clinicians (Webb et al. 2019). They addressed difficult prognostication and aggressive end-of-life care including use of transfusions in the final days of life. Amelia M. Breyre, MD, NREMT-P, and David H. Wang, MD, FAAHPM, presented on partnering with emergency medical services to improve serious illness care (Wang et al. 2023). Presenters addressed reasons for patients returning to the hospital from hospice and innovative ideas for collaboration with local emergency medical services to meet end-of-life needs. They also called for emergency medical services clinicians to receive end-of-life care training and the need for state/national policies and systems-based changes to build such partnerships.

In 2023, day 2 opened with a session on palliative justice for minoritized and marginalized peoples featuring Afsan Bhadelia, PhD, Katherine Bristowe, PhD, MA, BA (Hons), FHEA Debbie Braybrook, BSc, PhD, and Crystal Brown, MD, MA. Dr. Bhadelia opened the session discussing how healthcare has long failed to incorporate the values of marginalized and minoritized communities who have experienced structural violence and historical trauma (Bhadelia et al. 2022). Drs. Bristowe and Braybrook discussed creating LGBTQIA+ inclusive environments in palliative care, presenting on their ACCESSCare studies (Bristowe et al. 2018; Braybrook et al. 2023). Dr. Brown followed by highlighting key points of a qualitative study exploring Black patients’ experience of racism in serious illness through patient–clinician communication and medical decision-making within a racialized health care setting (Brown et al. 2023).

In 2024, day 2 opened with the editors of the textbook *Intentionally Interprofessional Palliative Care* (Donesky et al. 2024), presenting a lens through which all team members approach high-quality holistic palliative care at every level. DorAnne Donesky, PhD ANP-BC ACHPN FHPN, Michelle Marie Milic, MD, Naomi Saks, MA, Mdiv, BCC, and Cara L. Wallace, PhD, LMSW, APHSW-C, spoke to their specific professional roles while also emphasizing how intentionally interprofessional palliative care engages all members to perform at the top of their education and training and involves full team screening and intervention across domains.

The Palliative Story Exchange - a virtual gathering space for clinicians to share and reflect on their own lived experiences - was featured in 2023 and 2024 (Drutchas et al. 2024). Alexis E. Drutchas, MD, Richard E. Leiter, MD, MA, and Rachel Rusch, MSW, LCSW, MA, APHSW-C, invited conference participants to share a personal story on something that has moved them in recent months, be it at work or at home. They asked volunteers to speak about their own lived experience rather than traditional “patient cases.” All volunteers exhibited courage and vulnerability sharing moving stories that elicited topics of suffering, loss, grief, and hope.

In 2024, an interprofessional team from the University of California, San Francisco provided a thoughtful and emotional discussion of a Buddhist man at the end of life in the intensive care unit for the special Visiting Virtual Schwartz Center Rounds. Brian Block, MD, started the session with an overview of the case and his role as an intensivist. Catherine Saiki, RN, MSN, AGNP-C, ACHPN, spoke on her role as the hospice nurse practitioner, and Annabelle Bull, BSN, RN, CHPN, described her experience as the bedside nurse caring for a dying patient. Enver Rahmanov, MTS, MA, spoke to the spiritual and religious components of the case, while Kiran Gupta, MD, MPH, moderated the session. All panelists spoke to the human dimension of caring for the patient, sharing their vulnerabilities, feelings, and insights.

Day 2 for both years also featured 2 unique breakout sessions. In 2023, one session was on serious illness communication and another on palliative care for end-stage non-cancer conditions. In the communication breakout, Shunichi Nakagawa, MD, opened by sharing the expansion of VitalTalk in Japan, called “KanwaTalk” (Onishi et al. 2021). Justin Sanders, MD, MSc, followed with a presentation on the development and implementation of a structured guide to support serious illness conversations between oncologists and Black patients (Sanders et al. 2023). Kristin Levoy, PhD, MSN, RN, OCN, CNE, concluded the breakout with results from a meta-analysis of advance care planning and end-of-life cancer care (Levoy et al. 2023). She reviewed the current debates in the field on the value of advance care planning and discussed outcome measures for future research. In the concurrent break-out session on end-stage non-cancer conditions, Shelli Feder, PhD, APRN, FNP-C, ACHPN, presented the state of evidence for palliative care in heart failure, including results of a retrospective study examining palliative care referrals in veterans with heart failure (Feder et al. 2024). Donald Sullivan, MD, MA, MCR, followed with a presentation on palliative care for serious respiratory conditions, highlighting the quality of life impairments patients suffer and the impact of palliative care on symptoms and end-of-life care (Sullivan et al. 2022). Cristal Brown, MD, MHS, concluded the session on palliative care in advanced liver disease, including approaches to physical symptom management, psychosocial and emotional support, and communication (Brown et al. 2024).

The 2024 breakout sessions featured Rabia S. Atayee, PharmD, BCPS, APh, FAAHPM, Karen Bullock, PhD, LICSW, APHSW-C, Noelle Marie C. Javier, MD, Masako Mayahara, PhD, RN, CHPN, FPCN, FAAN, and Reverend Ramona Winfield, MDiv, BCC, who spoke on leadership experiences as women of color in hospice and palliative care. Sonia Malhotra, MD, MS, FAAP, FAAHPM, moderated the presentations and discussion. Panelists discussed intersectional identities, what has impacted them most as women of color, racism and discrimination in the field, and how they practice advocacy and resilience. The other concurrent breakout session in 2024 featured Laveena Munshi, MD, MSc, Judith Nelson, MD, JD, and Naveen Salins, MD, PhD, FRCP, on the topic of potentially non-beneficial interventions at the end of life (Salins et al. 2024). Dr. Munshi reviewed data on cardio-pulmonary resuscitation at the end-of-life (e.g., frequency and outcomes) and explored clinician and patient beliefs about this intervention (Chawla et al. 2023). Panelists also discussed the role of ethics and other approaches to managing discordance between clinicians and patients/families along with the emotional impact these discussions have on clinicians.

Both years featured a 70-min session of 5 rapid-fire talks from diverse palliative care specialists and researchers. In 2023, the theme of presentations focused on bringing together compassionate communities. Tracy Fasolino, PhD, APRN, spoke on expanding access to palliative care in rural Appalachia by applying an embedded palliative care model (Fasolino et al. 2024). Aileen Collier, BSc (Hons), PhD, discussed qualitative research on the use of telehealth in Australia and New Zealand while calling out structural inequity regarding access to technology and quality care in rural areas (Collier et al. 2016). Conference attendees learned about palliative care access in Malaysia from a study led by Malar Velli Segarmurthy, MD, MPH, who described the stark obstacles for Malaysians living in rural areas and opportunities for decentralization of palliative care (Segarmurthy et al. 2024). Alison Wiesenthal, MD, shared expertise on military palliative care in the USA, advantages of concurrent hospice care, combat palliative care, and opportunities for support. Rachel Thienprayoon, MD, MSCS, concluded on organizational compassion in healthcare and ways to operationalize this concept across various settings, within a team and health systems (Thienprayoon 2023).

In 2024, the rapid-fire session theme was “palliative care access and quality.” Daniel Shalev, MD, described the prevalence of mental health comorbidities, identified barriers for palliative care clinicians to provide mental healthcare, and provided systems-based goals for mental health integration into palliative care (Wozniak et al. 2021). Amanda Kastrinos, PhD, followed with a discussion of the “hidden” caregiving generation: young adult caregivers and how the experience impacts their developmental trajectory and mental health (Kastrinos et al. 2022). She presented a novel, tailored young adult caregiving intervention to initiate difficult family conversations and reduce patient and family distress. Next, Cristiane Bergerot, PhD, presented on the value of geriatric assessment for older adults with metastatic cancer undergoing active treatment in a severely resource-constrained setting (Dale et al. 2023). Megan R. Miller, PhD, RN, then followed with a presentation on her mixed methods research examining relationships between spirituality, pain, and pain-related catastrophizing among patients with cancer receiving active treatment (Miller et al. 2023). Lastly, Columba Thomas, MD, OP, closed the session with a discussion of palliative sedation at the end of life. He described its role in managing delirium and existential suffering, while detailing gaps in the research and sharing approaches to overcome challenges with sedation at the end of life (Thomas et al. 2024).

For both years, the conference concluded with a closing keynote in honor of Dr. Richard Payne, former Chief of the Pain and Palliative Care Service at MSK on the application of principles of diversity, equity, and inclusion in palliative care. In 2023, Corey L. Kennard, MACM, spoke on 3 “silent screams” of patients: how people want to understand, to be understood, and to be heard. He remarked on the need for clinicians to understand patients as people beyond their medical diagnoses, discussed cultural influences in healthcare, and shared approaches to culturally appropriate communication. He called on clinicians to build trust with patients by exploring spiritual beliefs, stating “trust is a human-to-human experience, not a patient-to-healthcare worker experience.” In 2024, Sonia Malhotra, MD, MS, FAAP, FAAHPM, described the intersectionality of culture, race, and spirituality while describing the impact of racial disparities on access and quality of healthcare. She told a personal story of advocating for her father to receive end-of-life care that met his wishes and aligned with his cultural values. Dr. Malhotra described approaches to build trust not only with patients and their families but with each other as clinicians. She called on the need to do better, asking, “Do we have to burn others to see the light?” and provided strategies to become an ally for minoritized patients, families, and colleagues.

## Outcomes

Conference CME evaluations were collected and reviewed for each year. The post-conference evaluation was completed by 30% of attendees in 2023 (*n* = 229 responses of 763 registrants) and 24% in 2024 (*n* = 423 responses of 1729 registrants). Professionals represented included nurses, nurse practitioners, social workers, physicians, physician assistants, chaplains, pharmacists, physical therapists, occupational therapists, psychologists, mental health counselors, integrative medicine practitioners, students, and administrative and research personnel working in various clinical and academic settings, including medical or acute care units, outpatient clinics, long-term care, inpatient and home hospices, inpatient palliative care units, community-based and home care services, and academic and university settings.

In 2023 and 2024, most respondents (86% and 85%, respectively) identified as a clinician who manages or cares for patients, with the largest representation from nursing professionals (50% in 2023 and 56% in 2024). Most respondents rated the overall format and length of the program as good or excellent (100% and 99%, respectively, for both years). Most respondents (93% in 2023 and 92% in 2024) thought the activity incorporated principles of equity, diversity, and inclusion to the extent possible and most (86% in 2023 and 89% in 2024) reported the conference supported interprofessional care practice. Most respondents (93% in 2023 and 92% in 2024) reported being very satisfied with the CME activity overall (scored ≥8 on a 0–10 scale). Participants commented, “It was the most heartfelt of any CME activity I’ve attended,” and “The mix of science and humanism moved me,” in addition to repetitive acknowledgment of the importance of free registration.

Participants committed to change in areas of patient and interprofessional communication, patient education, teamwork roles and responsibilities, treatment, quality improvement, diagnosis and screening, and safety, among other areas. Some specific examples from comments included: [I will]

- “utilize a lens of trauma-informed care to provide high quality communication and care for my patients”;
- “look for opportunities to have conversation[s] about innovation in the implementation of patient-centered, culturally concordant care practices”;
- “focus on increasing my use of non-pharmacologic interventions for help with symptom management with the goal of improving my patient’s quality of life”; and
- “remember that all people are fighting a battle and that even clinicians are personally affected by the issues and illnesses that we see every day in our patients.”

Most respondents (95% in 2923 and 97% in 2024) reported the conference will help them better engage with other members of the interprofessional team in the care of patients. Examples included: “[I will] recognize the distinction between multidisciplinary versus interprofessional communication,” and “[I will] continue to advocate for more intentional interprofessional communication and express my appreciation for everyone’s role.”

## Conclusion

Post-conference evaluations highlighted the unique contribution of our MSK conference to the hospice and palliative care field with an emphasis on global and multicultural inclusion, interprofessional and collaborative practice, social justice, and both art and science (Rosa et al. 2021, 2022c, 2023b). We maintain commitments to ensuring diversity in our planning committee and among invited faculty, as well as equitable program access through continued complimentary registration and staggered scheduling to support live attendance across time zones. The 6^th^ Annual U.S. Celebration of WHPCD will be a 2-day virtual event held on October 6–7, 2025, continuing a successful and well-received format. We anticipate that our annual WHPCD conference will continue to serve as a safe, inclusive space for the global hospice and palliative care field for years to come.
